# Hotel Life

**Published:** 1860-01

**Authors:** 


### HOTEL LIFE.

Of all the miserable ways of living, that of hotels and boarding-houses takes the lead. One of the best sermons we ever heard, as connected with domestic life, was delivered in New-Orleans many years ago, by that eminent divine and scholar, the Rev. W. A. Scott, D.D., now of San Francisco. We thought, at the time, that humanity would have been a gainer, if a tract had been made of it and placed in the hands of every married couple in the Union. It is hoped that, should the eminent author ever see this article, he will publish the pith of it in his own monthly. A life of this sort eats out domestic love; it creates a morbid desire for tinsel and show; it cultivates sham in morals, in dress, in personal deportment; it turns every thing into pretense and hollowness. There is no depth in any thing that is really useful or good. All is superficial, cold, and heartless.

From such a life, gormandizing, idleness, and *ennui* are inseparable; eating, sleeping, lounging, and dilettauteing make the dreary routine—the two great events of each succeeding day being the dinner and the opera or theatre or lecture. They wake to think of what there shall be for breakfast, and, after reading the morning papers and an objectless and lazy stroll, the subject of conjecture or conversation, if not both, is what kind of dinner will be spread; if this or that new or rare or favorite dish will be in the bill of fare.

As to conversation, there can be no real intercommunion with persons whose acquaintance rarely exceeds a month, oftener not a week. There is nothing to draw out the better natures and the deeper feelings of the heart in a transient "society" like this, while the risk of becoming acquainted with unworthy persons is very great. To young sons and daughters it is simply fearful, for adventurers, fortune-hunters, and pretenders, with fast young men and those who have nothing but a fine personal appearance, are the *habitues* of all public places.

But there are physical evils of the most serious nature. When a wife or daughter has nothing to do, and the appetite is stimulated day after day by all the arts of "scientific cookery," when the five o'clock dinner is universal, and when the stomach is "raving" for food in consequence of the almost entire abstinence since breakfast, a double work is thrown in upon it in its debilitated state, and keeps it "laboring" during the greater part of the night, making what ought to be the hours of peaceful rest, absolutely hideous by terrible dreams, and the morning comes without the blest renewal of strength which healthful sleep would have given, and this for weeks and months together. Verily, it is no wonder that the thoughtful physician should apply the epithet, "Thou fool," to any parent who would expose a family to such a life. And in the light of it, we may gather that the most certain means of making life a failure *in toto* on the part of any newly-married couple, is to "go to boarding." Better a thousand times, socially, morally, and physically, hire a two-roomed shanty, live on bread and potatoes and do the housework without the aid of menials, and continue to do these things until means are accumulated to take a step higher. Thus doing, we would not see a tithe of the sick wives we now do, not a tithe of the unhappy matches, the disgraceful divorces and the early wreck of business prospects which leave so many men disabled before they are thirty years of age; disabled for life from engaging in any handsomely profitable employment in consequence of a load of indebtedness which it would take a lifetime to liquidate.

In view of these things, our advice to every young man of energy, a high spirit, and any respectable calling, is, marry before you are thirty, even if you have not five dollars ahead. Take a cabin of a single room, if you can do no better; live within your means, whatever Mrs. Grundy may say, and with moderate perseverance, never rising faster than your gains, things will go well with you, and three times out of four you will, in a race of twenty years, come out triumphantly ahead of those who had a small fortune to begin with — theirs having insensibly dwindled away, while yours is increasing with a steady and wholesome rapidity.

